# METTL3-dependent DLG2 inhibits the malignant progression of cervical cancer by inactivating the Hippo/YAP signaling

**DOI:** 10.1186/s41065-025-00365-z

**Published:** 2025-01-25

**Authors:** Mei Pu, Xia Xiao, Shasha Lv, Daqing Ran, Qian Huang, Mingming Zhou, Qirong Lei, Lingshuang Kong, Qing Zhang

**Affiliations:** 1 Department of Obstetrics and Gynecology, Dazhou Vocational and Technical College, Dazhou, 635001 China; 2 Chongqing Key Laboratory of Translational Research for Cancer Metastasis and Individualized Treatment, Chongqing University Cancer Hospital & Chongqing Cancer Institute & Chongqing Cancer Hospital, No. 181 Hanyu Road, Shapingba District, Chongqing, 400030 China; 3 Department of Dermatology, The Affiliated Hospital of Southwest Medical University, No. 25 Taiping Street, Jiangyang District, Luzhou, 646000 China; 4 Department of Nursing, The Affiliated Hospital of Southwest Medical University, No. 25 Taiping Street, Jiangyang District, Luzhou, 646000 China

**Keywords:** Cervical cancer, DLG2, METTL3, Hippo/YAP signaling pathway

## Abstract

**Background:**

Discs large homolog 2 (DLG2) has been implicated in cancer development, yet its role in cervical cancer remains unclear. This study aims to explore the regulatory mechanism of DLG2 in cervical cancer and its clinical implications.

**Methods:**

Quantitative reverse transcription polymerase chain reaction and western blotting assays were employed to detect RNA and protein expression, respectively. Colony formation assay, 5-Ethynyl-2’-deoxyuridine assay, flow cytometry, and transwell assays were conducted for cell functional analysis. A xenograft mouse model assay was performed to analyze tumor tumorigenesis in vivo. m6A RNA immunoprecipitation assay was used to analyze the association of METTL3 and DLG2.

**Results:**

DLG2 was underexpressed in cervical cancer tissues and cells. Elevating DLG2 levels significantly suppressed cervical cancer cell proliferation, migration, and invasion, while promoting apoptosis. Additionally, DLG2 overexpression led to the deactivation of the Hippo/YAP signaling pathway. In vivo, DLG2 overexpression was shown to reduce tumor formation. We also discovered that METTL3 destabilized DLG2 mRNA through an m6A-dependent mechanism. Moreover, lowering DLG2 expression mitigated the effects of METTL3 silencing on cervical cancer cell malignancy.

**Conclusion:**

DLG2 acted as a tumor suppressor in cervical cancer by inhibiting the Hippo/YAP signaling pathway. The METTL3-dependent regulation of DLG2 mRNA stability could be a critical factor in cervical cancer progression.

**Supplementary Information:**

The online version contains supplementary material available at 10.1186/s41065-025-00365-z.

## Introduction

The incidence rate of cervical cancer ranks fourth among female malignant tumors [1], and persistent human papillomavirus (HPV) infection is a major factor in its progression [2, 3]. HPV vaccination is a primary method for the prevention of cervical cancer [4]. The traditional treatment methods for cervical cancer patients usually include surgical resection, radiotherapy, and chemotherapy, with specific schemes varying according to clinical pathological conditions such as tumor staging [5, 6]. In recent years, targeted treatment drugs against specific molecules have significantly improved the treatment outcomes for some patients [7, 8], indicating that studying the molecular mechanisms of cervical cancer development is important for the treatment of this disease.

The Hippo pathway is a tissue growth regulator that plays a crucial role in organ development and regeneration [9]. This pathway consists of a kinase module including large tumor suppressor 1 (LATS1) and a transcription module containing PDZ-binding motif (TAZ) and the Yes-associated protein (YAP) [10]. Upon activation of the Hippo signaling pathway, MST1/2, as the core component of this kinase chain, is first activated, leading to the phosphorylation of downstream genes LATS1/2. LATS1/2 primarily inhibits the proliferation and migration ability of tumor cells by blocking the cell cycle process and plays a significant regulatory role in mediating apoptosis. LATS1/2, as the upstream gene of YAP/TAZ, causes phosphorylation of both [11]. In many types of cancer, the activation of TAZ/YAP is associated with a variety of tumor-promoting functions [12]. The accumulating evidence revealed the potential of the Hippo pathway as the therapeutic target for cancers [13]. Thus, an in-depth investigation into the mechanism responsible for cervical cancer progression is necessary for the treatment of this disorder.

Studies suggest that Discs large homolog 2 (DLG2) gene is a regulator of the Hippo signaling pathway [24]. DLG2 is a member of the Discs large (DLG) family and can regulate subcellular polarity, thereby enhancing cellular adaptability to complete processes such as proliferation, apoptosis, stress adaptation, and organelle biological functions [14]. As reported by Keane et al., DLG2 alters cyclins A and B and regulates DNA damage to mediate the cell cycle [15]. In addition, this protein can induce cell death through the regulation of the Bax/Bcl2 [16]. The protein encoded by DLG2 is the postsynaptic scaffolding protein DLG2, which interacts with NMDA receptors, potassium channels, and cytoskeletal regulatory factors [17]. These interactions have a significant impact on neuronal signal transmission and synaptic plasticity. In different cellular environments, DLG2 can either inhibit tumor formation or promote tumorigenesis [18, 19]. However, there is limited evidence revealing its role and mechanism in cervical cancer progression.

Methyltransferase 3 (METTL3) has 580 amino acids and is an important component of the m6A methyltransferase complex, primarily responsible for catalyzing the m6A modification on the RNA molecule at the N6-methyladenosine site [20]. Furthermore, it can interact with translation initiation mechanisms other than methyltransferases and m6A methylation reading proteins [20]. It is commonly dysregulated in cancers and acts as an oncogene in acute myeloid leukemia, liver cancer and lung cancer or a tumor suppressor in bladder cancer [21]. In particular, METTL3 contributed to cervical cancer progression [22]. Although research has revealed that many genes are subject to RNA m6A modification and thus participate in tumor progression [23], to date, there have been no reports on the relationship between DLG2 and RNA m6A modification, or on whether DLG2 can influence the malignant phenotype of cervical cancer through this pathway.

Thus, we postulated that the METTL3/DLG2/Hippo signaling pathway was implicated in the progression of cervical cancer, and this hypothesis has been substantiated in the current work. The aim was to uncover the molecular mechanisms driving the progression of cervical cancer.

## Materials and methods

### Clinical samples

A portion of this study involved the use of human clinical specimens, which included the retrospective collection of 39 tissue samples from patients with cervical cancer at Chongqing University Cancer Hospital and 39 normal cervical tissue samples. All samples were stored at -80℃. Immunohistochemical staining was performed on these samples. The clinical pathology experiments related to this study were reviewed and approved by the Ethics Committee of Chongqing University Cancer Hospital, and written informed consent was obtained from the patients.

### Cell culture

Human cervical epithelial immortalized cell line End1/E6E7 was purchased from Yaji Biological (Shanghai, China) and cultured in RPMI-1640 (EK-Bioscience, Shanghai, China). Cervical cancer cells including SiHa and C33A were purchased from EK-Bioscience and maintained in DMEM (EK-Bioscience). These cells were cultured at 37˚C with 10% fetal bovine serum (EK-Bioscience), 1% penicillin/streptomycin (Millipore, Bradford, MA, USA), and 5% CO_2_.

### Cell transfection

DLG2 overexpression plasmid, METTL3 overexpression plasmid, and the small hairpin RNAs of METTL3 (sh-METTL3 5’-GATCCTCAAGGA AACATGCTGCCTCAAGAGAGGCAGCATGTTTCCTTGATTTTTG-3’ and 5’-AATTCAAAAATCAAGGA AACATGCTGCCTCACTTCAGGCAGCATGTTTCCTTGAG-3’) and DLG2 (sh-DLG2 5’-GATCCTAGCACAATATCAACCTGAACAAGAGTTCAGGTTGATATTGTGCTATTTTTG-3’ and 5’-AATTCAAAAATAGCACAATATCAACCTGAACACTTCTTCAGGTTGATATTGTGCTAG-3’) were provided by Ribobio Co., Ltd (Guangzhou, China). Cervical cancer cells were seeded into cell culture plates. shRNAs or plasmids were added to Opti-MEM (Thermo Fisher, Waltham, MA, USA), and the mixtures were gently swirled to ensure uniform distribution. Lipofectamine 3000 reagent (Thermo Fisher) was then added to the Opti-MEM solution containing siRNAs, and the mixtures were gently swirled again. The transfection mixture was added dropwise to the cell culture medium. Twelve to eighteen hours later, the cells were replenished with fresh medium and continued for subsequent experiments.

### Immunohistochemistry (IHC) assay

After deparaffinization and gradient alcohol dehydration, the paraffin tissue sections were subjected to antigen retrieval. The specimens were incubated with monoclonal antibodies against DLG2 (0.25 mg/mL, #34-4700, 1:200, Thermo Fisher) and METTL3 (1 mg/mL, #PA5-41599, 1:200, Thermo Fisher) at 4 °C. The secondary antibody (1 mg/mL, #S0001, 1:5000, Affinity, Nanjing, China) was added for 2 h. The slides were washed with phosphate buffer solution (PBS) three times. DAPI (Solarbio, Beijing, China) solution was added to stain the cell nuclei. Fluorescence microscopy was used to observe and photograph the slides. The assay was performed with three biological replicates.

### Quantitative real-time polymerase chain reaction (qRT-PCR)

Total RNA was isolated using Trizol reagent (TaKaRa, Shiga, Japan). In brief, cell and tissue samples were exposed to Trizol reagent to ensure complete lysis. Chloroform was added to the lysates, and the upper aqueous phase was transferred to RNase-free tubes, followed by the addition of isopropyl alcohol and 75% ethanol. The RNA pellets were dissolved in RNase-free water. The concentration of RNA was measured using a spectrophotometer (Thermo Fisher). The ratio of OD260/OD280 was found to be between 1.8 and 2.0. According to the manufacturer’s instructions, a 10 µL reaction mix without DNA (Thermo Fisher) was prepared, followed by incubation at 4 °C. Subsequently, a 20 µL reverse transcription reaction mix (Thermo Fisher) was incubated at 37 °C for 15 min. Quantitative PCR was conducted using SYBR reagents (TaKaRa) in ABI StepOne qRT-PCR System (Thermo Fisher). The qRT-PCR was cycled at 95 °C for 20 s, 40 cycles at 95 °C for 3 s, and 60 °C for 20 s. The data were analyzed using the 2^−ΔΔCt^ method. Primer sequences are shown in Table 1. The assay was performed with three biological replicates for cell samples and with 39 biological replicates for clinical tumor samples.

**Table 1:** Primer sequences used in qRT-PCR

Name	Primers for qRT-PCR (5’-3’)	
METTL3	Forward	ATCCCCAAGGCTTCAACCAG
	Reverse	GCGAGTGCCAGGAGATAGTC
DLG2	Forward	ACTGAGGGGAAGACTCAGC
	Reverse	GGCCCACCTGTCTTCTTGAC
LATS1	Forward	ATACTTGGGGTTGCTGGGAC
	Reverse	AGGAAGTCCCCAGGACTGTT
YAP1	Forward	TCCCAGATGAACGTCACAGC
	Reverse	AGGGTCAAGCCTTGGGTCTA
TAZ	Forward	TGGACCAAGTACATGAACCACC
	Reverse	CTGGTGATTGGACACGGTGA
β-actin	Forward	CTTCGCGGGCGACGAT
	Reverse	CCACATAGGAATCCTTCTGACC

### Western blotting assay

Cell and tumor samples were subjected to lysis using radioimmunoprecipitation assay (RIPA) buffer (Beyotime, Shanghai, China) containing PMSF as a phosphatase inhibitor. The mixtures were heated at 100 °C for 5 min. Subsequently, the protein concentration was measured through a bicinchoninic acid assay (BCA) protein assay kit (Beyotime). The proteins were transferred to PVDF membranes, blocked with 5% non-fat milk, and incubated with primary antibodies against DLG2 (0.25 mg/mL, #34-4700, 1:200, Thermo Fisher), LATS1 (0.67 mg/mL, #PA5-78278, 1:2000, Thermo Fisher), YAP1 (1.0 mg/mL, #PA1-46189, 1:1000, Thermo Fisher), p-YAP1 (59.2 µg/mL, #PA5-17481, 1:1000, Thermo Fisher), TAZ (0.5 mg/mL, #703032, 1:5000, Thermo Fisher), p-TAZ (#AF4315, 1:1000, Affinity), METTL3 (1 mg/mL, #PA5-41599, 1:200, Thermo Fisher), and β-actin (#AF7018, 1:5000, Affinity) at 4 °C overnight. The following day, the secondary antibody (1 mg/mL, #S0001, 1:5000, Affinity) was added and the membranes were incubated for 1 h. Finally, the blots were visualized using the ECL reagent (Beyotime). The assay was performed with three biological replicates.

### Cell colony formation assay

Well-grown cervical cancer cells were selected and counted, followed by adding to 6-well plates (300 cells per well). The culture medium was supplemented to 2 mL and changed every two days. When cell colonies had grown to an appropriate size, the medium was discarded. After being rinsed with PBS, the cells were stained using 0.1% crystal violet. The 6-well plates were placed under white light, and images were captured using a camera. The assay was performed with three biological replicates.

### 5-Ethynyl-2’-deoxyuridine (EdU) assay

The EdU Imaging Kit (Guangzhou Ribo Biotechnology Co., Ltd., Guangzhou, China) was used for a cell proliferation experiment. Following the instructions of the kit, the cells were labeled with EdU. Capture and enumeration of migrated or invasive cells were conducted by selecting five random fields of view under an inverted microscope for photography and counting purposes. The assay was performed with three biological replicates.

### Cell apoptosis analysis

Forty-eight hours after transfection, the cells were collected with 0.25% trypsin. Five µL of Annexin V-FITC and 5 µL of PI were added to the cells, and the mixture was stained in the dark for 15 min. Finally, the stained cells were analyzed using an AttuneTM NxT flow cytometer (Thermo Fisher). The assay was performed with three biological replicates.

### Transwell assays

Matrigel (Abwbio, Shanghai, China) was diluted with DMEM (EK-Bioscience), and then 60 µL of the diluted solution was added to each Transwell chamber for cell invasion assay. The chambers were placed in a humidified incubator with 5% CO_2_ to allow the Matrigel to solidify. Cervical cancer cells were collected and suspended in DMEM medium (without FBS) to create a single-cell suspension, which was then counted and adjusted to an appropriate concentration. Complete DMEM medium with 10% FBS was added to the lower chambers of the Transwell and the chambers were inserted. Cells were inoculated at 1.5 × 10^5^ cells per chamber in the upper Transwell chambers. The chambers were placed back into the cell culture incubator for continued cultivation. After 24 h, the chambers were fixed with paraformaldehyde and stained with 0.1% crystal violet solution for 20 min. The bottom of the chambers was carefully removed with a scalpel, mounted on glass slides with neutral resin, and air-dried. Five random fields of view were selected under an inverted microscope for photography and counting of the migrated or invading cells. The assay was performed with three biological replicates.

### Xenograft mouse model assay

Two groups of nude mouse subcutaneous tumor experiments were conducted, with each group consisting of 5 female nude mice (5 weeks of age, Hunan Slyke Jingda Experimental Animal Co., LTD., Changsha, China). SiHa cells stably expressing DLG2 or Vector were cultured, and the cell suspensions were centrifuged. The supernatant was discarded, and the cell pellets were retained. A total of 200 µL of cell suspension containing 5 × 10^6^ cells was injected into the subcutaneous tissue of the armpit of the nude mouse, which was then marked for identification. The status of the nude mice and the growth of the tumors in the armpit were regularly observed. The nude mice were euthanized, and the tumors were excised from the armpit and subjected to immunohistochemical staining, western blotting analysis, and image capture. The Animal Care and Use Committee of Chongqing University Cancer Hospital approved the study. The assay was performed with five biological replicates.

### m6A RNA immunoprecipitation (MeRIP) assay

RNA-antibody (m6A antibody, 1 mg/mL, #ab208577) complexes that had been incubated overnight were added to the pre-treated protein A/G magnetic beads (BersinBio, Guangzhou, China) and rotated on a 4 °C shaker for 2 h. The magnetic beads were collected on the magnetic stand. Proteinase K digestion solution (Roche, Basel, Switzerland) was used to resuspend the RIP products, which were then placed in a magnetic rack to collect magnetic beads. Brief centrifugation was performed to pellet the supernatant, which was transferred to enzyme-free centrifuge tubes. A mixture of phenol, chloroform and isoamyl alcohol was added to the tubes and centrifuged to collect the upper water phase. The samples were incubated with glycogen, sodium acetate and ethanol. The RNA pellets were resolved in RNase-free water, and qRT-PCR was used to analyze the expression level of DLG2. The assay was performed with three biological replicates.

### RNA stability assay

Cell cultures were inoculated into corresponding wells of 6-well plates. After overnight incubation, the culture medium was replaced with a complete culture medium containing 2 µg/mL actinomycin D (Rechemscience, Shang, China). The cells were then incubated for 0 h, 2 h, 4 h, or 6 h. After the specified incubation times, total RNA was extracted, and the relative expression level of DLG2 mRNA was detected using qRT-PCR methodology. The assay was performed with three biological replicates.

### Statistical analysis

Statistical analysis was conducted using GraphPad Prism 8.0. Gene expression analysis in paired tissues involved the use of paired *t*-tests to compare differences between groups. Experimental data from two groups were represented as mean ± standard deviation (SD), and differences were analyzed using unpaired *t*-tests, one-way ANOVA, and two-way ANOVA tests. For all analyses, *P* less than 0.05 indicated statistical significance.

## Results

### DLG2 expression was downregulated in cervical cancer tissues and cells

**Fig. 1:**
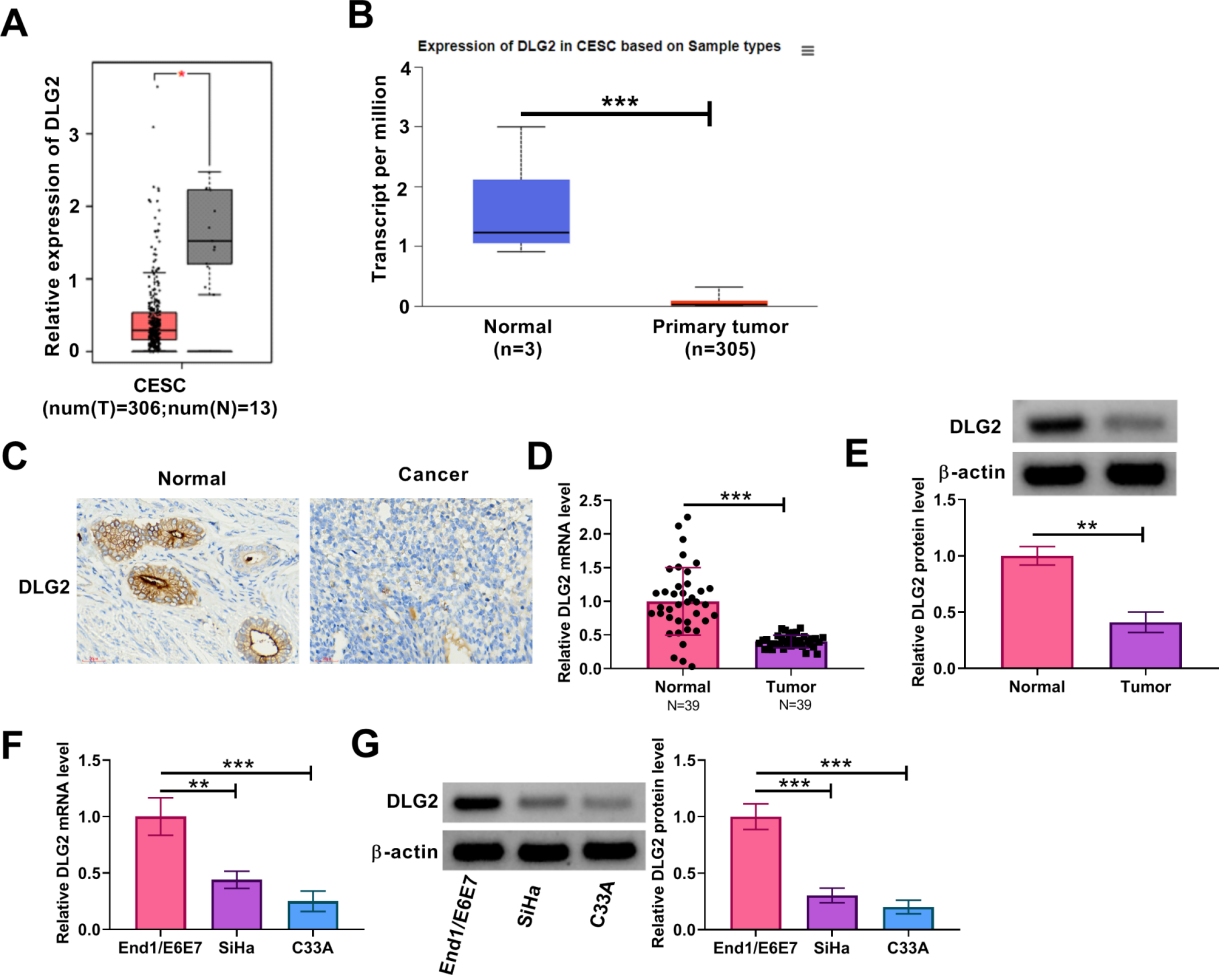
DLG2 expression was downregulated in cervical cancer tissues and cells. (**A** and **B**) DLG2 expression in cervical cancer tissues (*N* = 306) and normal cervical tissues (*N* = 13) was analyzed through the GEPIA online database. (**B**) DLG2 expression in cervical cancer tissues and normal cervical tissues was analyzed through the UALCAN online database. (**C**) The IHC assay was performed to analyze DLG2 protein expression in cervical cancer tissues and normal cervical tissues. (**D**) DLG2 mRNA expression was detected by qRT-PCR in cervical cancer tissues (*N* = 39) and normal cervical tissues (*N* = 39). (**E**) DLG2 protein expression was analyzed by western blotting assay in cervical cancer tissues and normal cervical tissues. (**F**) DLG2 mRNA expression was detected by qRT-PCR in End1/E6E7 cells, SiHa cells, and C33A cells. (**G**) DLG2 protein expression was analyzed by western blotting assay in End1/E6E7 cells, SiHa cells, and C33A cells. CESC, cervical squamous cell carcinoma. *P < 0.05, **P < 0.01, and ***P < 0.001

We analyzed DLG2 expression in cervical cancer tissues and normal cervical tissues through the online databases including GEPIA and UALCAN. As shown in Fig. 1A and B, its expression was significantly downregulated in tumor tissues when compared with normal tissues. The IHC assay also showed its low expression in cervical cancer tissues in comparison with normal cervical tissues (Fig. 1C). The analysis of clinical tissue samples showed that DLG2 expression at mRNA and protein levels was lower in cervical cancer tissues than in normal cervical tissues (Fig. 1D and E). Moreover, the mRNA and protein levels of DLG2 were downregulated in cervical cancer cells (SiHa and C33A) when compared with human cervical endometrial epithelial cells (End1/E6E7), as shown in Fig. 1F and G. Thus, these data demonstrate that DLG2 expression is downregulated in cervical cancer tissues and cells.

### DLG2 overexpression inhibited cervical cancer cell proliferation, migration, and invasion and induced cell apoptosis

**Fig. 2:**
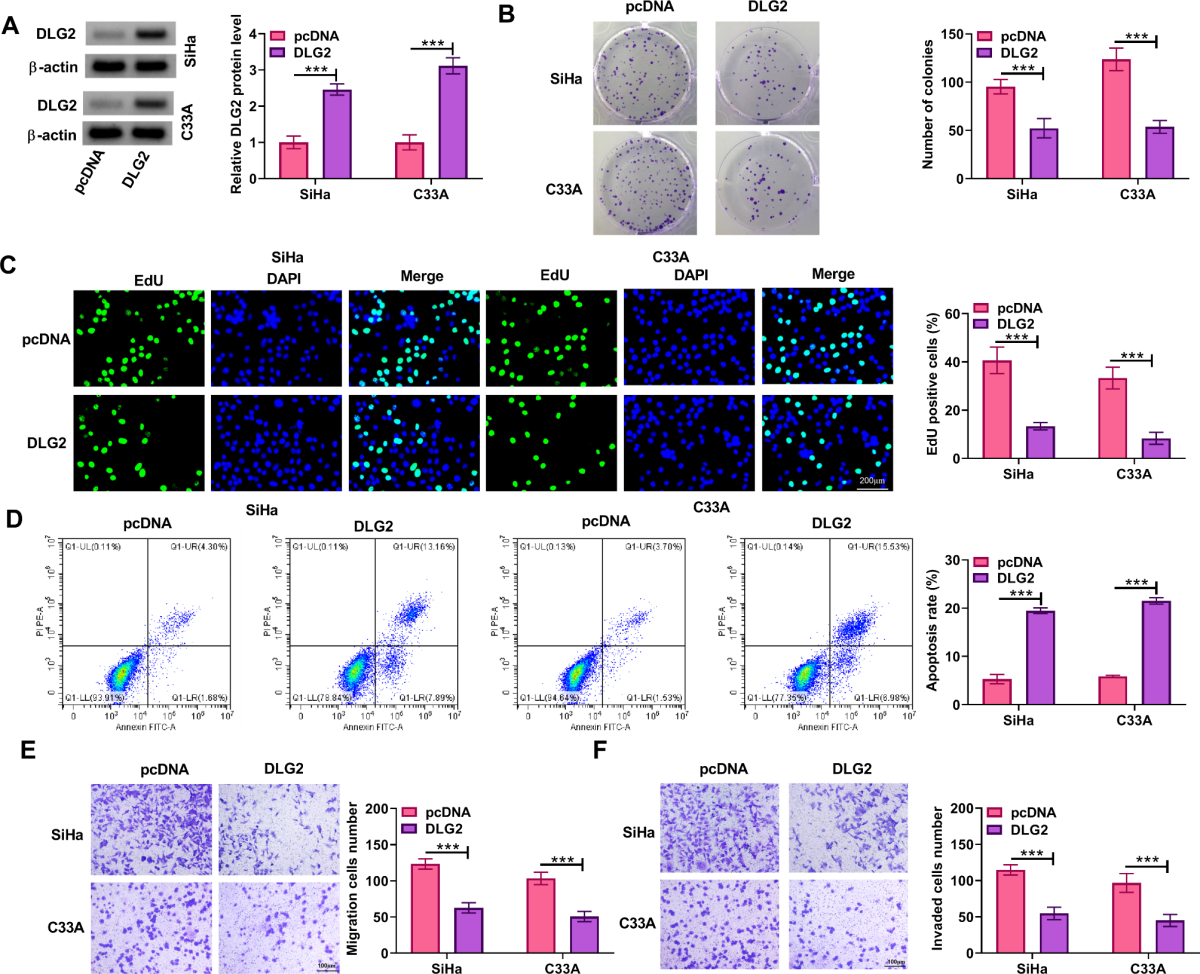
DLG2 overexpression inhibited cervical cancer cell proliferation, migration and invasion and induced cell apoptosis. Both SiHa and C33A cells were transfected with pcDNA or DLG2 overexpression plasmid. (**A**) DLG2 protein expression was detected by western blotting assay. (**B** and **C**) Cell proliferation was analyzed by cell colony formation assay and EdU assay. (**D**) Cell apoptosis was analyzed by flow cytometry. (**E** and **F**) Cell migration and invasion were assessed by transwell assays. ***P < 0.001

We then analyzed the effects of DLG2 on the malignant phenotypes of cervical cancer cells, including cell proliferation, apoptosis, migration, and invasion. The data in Fig. 2A indicated the successful overexpression of DLG2 in both SiHa and C33A cells. Subsequently, DLG2 overexpression reduced the number of positive colonies and EdU-positive cells (Fig. 2B and C). Moreover, the results showed that DLG2 overexpression increased cell apoptotic rate and reduced the number of migrated cells and invaded cells (Fig. 2D-F). Thus, DLG2 inhibited cervical cancer cell proliferation, migration, and invasion and promoted cell apoptosis.

### DLG2 overexpression inactivated the Hippo/YAP signaling in cervical cancer cells

**Fig. 3:**
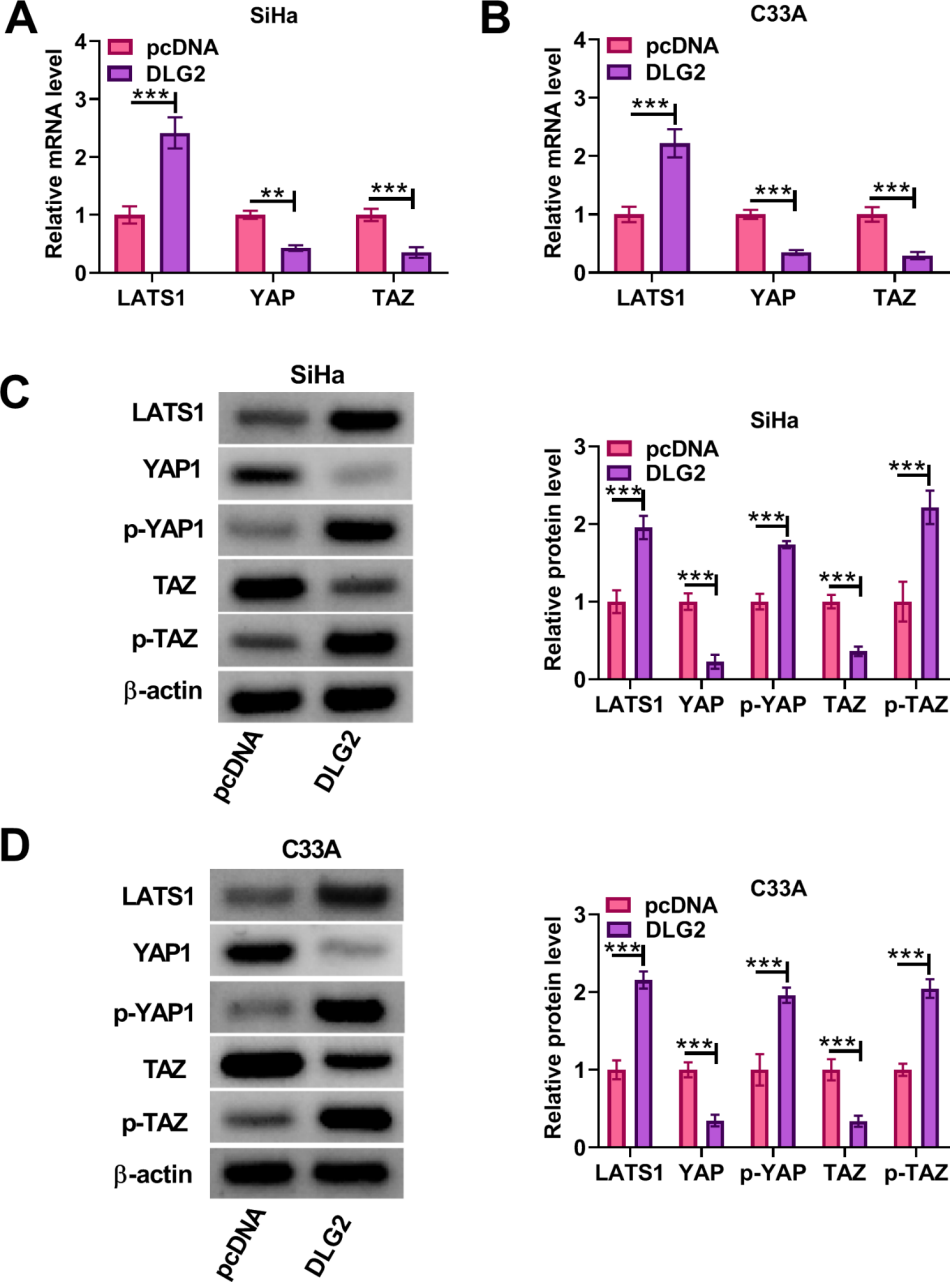
DLG2 overexpression inactivated the Hippo/YAP signaling in cervical cancer cells. Both SiHa and C33A cells were transfected with pcDNA or DLG2 overexpression plasmid. (**A** and **B**) The mRNA levels of LATS1, YAP, and TAZ were detected by qRT-PCR. (**C** and **D**) The protein levels of LATS1, YAP1, p-YAP, TAZ, and p-TAZ were analyzed by western blotting assays. **P < 0.01, and ***P < 0.001

We continued to investigate the effect of DLG2 overexpression on the Hippo/YAP signaling in both SiHa and C33A cells. To achieve this, we transfected DLG2 overexpression plasmid and negative control pcDNA into both SiHa and C33A cells. When the inhibitory Hippo kinase module is “turned on”, LATS1 and LATS2 phosphorylate YAP and TAZ, rendering them inactive [24]. Thus, we analyzed LATS1, YAP, TAZ, phosphorylated YAP, and phosphorylated TAZ in DLG2-overexpressing cells. The results showed that the ectopic DLG2 expression increased LATS1 mRNA expression but decreased YAP and TAZ mRNA levels in the cells (Fig. 3A and B). Moreover, DLG2 overexpression led to increases in the protein expression of LATS1, p-YAP and p-TAZ and decreases in the protein expression of YAP and TAZ in both SiHa and C33A cells (Fig. 3C and D). Thus, DLG2 could inactivate the Hippo/YAP signaling in cervical cancer cells.

### DLG2 overexpression inhibited tumor formation in vivo

**Fig. 4:**
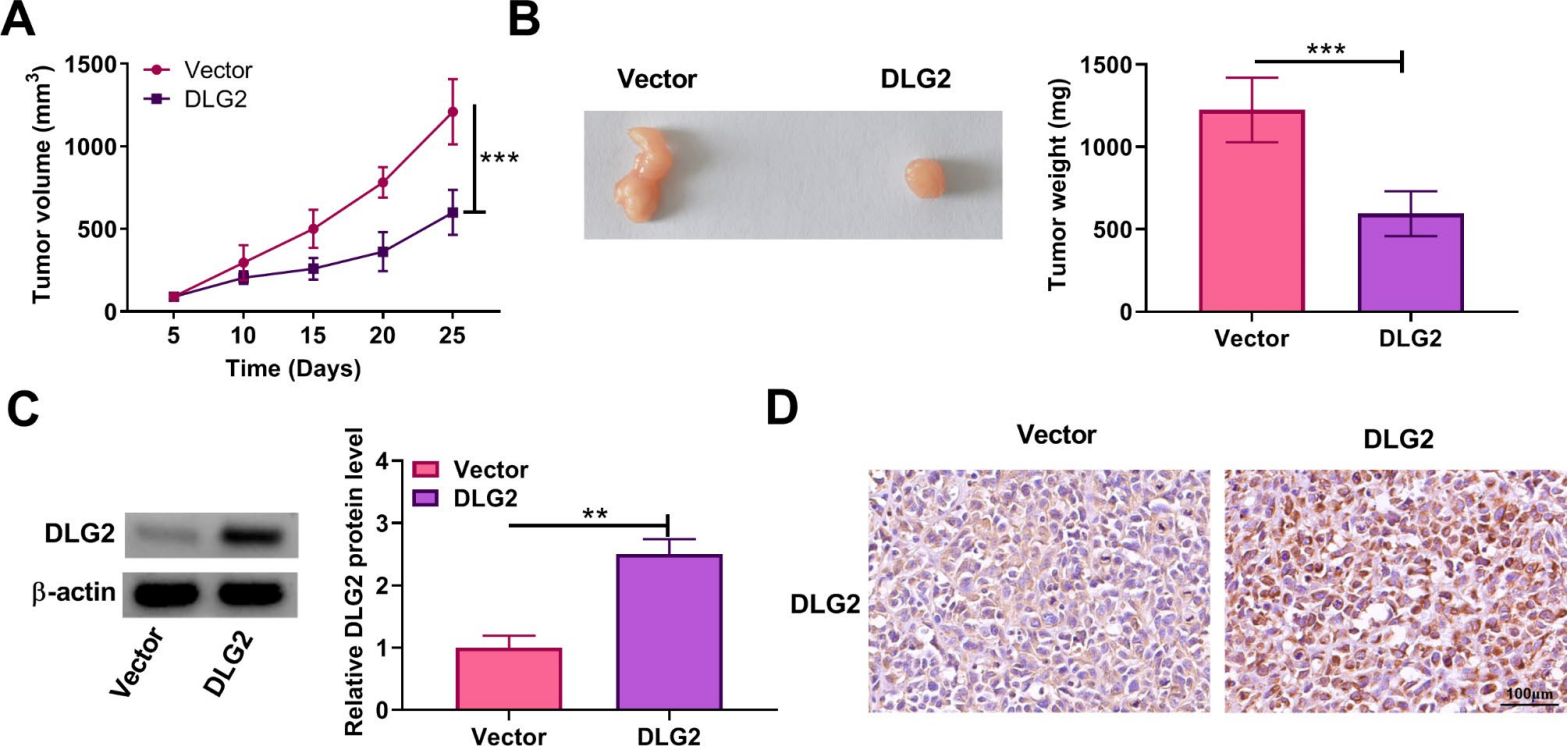
DLG2 overexpression inhibited tumor formationin vivo. SiHa cells stably expressing DLG2 or vector were injected into nude mice and tumor volume was analyzed every 5 days after 5 days of injection (**A**). After 25 days, the mice were euthanized and the forming tumors were harvested for tumor weight (**B**) and DLG2 protein expression analysis (**C** and **D**). **P < 0.01, and ***P < 0.001

We also analyzed the effect of DLG2 overexpression on tumor tumorigenesis through a xenograft mouse model assay. The model was established by injecting SiHa cells stably expressing DLG2 or vector into nude mice. The results showed that the treatment with DLG2 overexpression led to a delayed growth of tumors and a decrease in tumor weight (Fig. 4A and B). As shown in Fig. 4C and D, DLG2 protein expression was upregulated in the tumors resulting from SiHa cells stably expressing DLG2 when compared with those from the control group. Thus, DLG2 might inhibit the malignant growth of cervical cancer cells in vivo.

### METTL3 destabilized DLG2 mRNA expression in an m6A-dependent manner

**Fig. 5:**
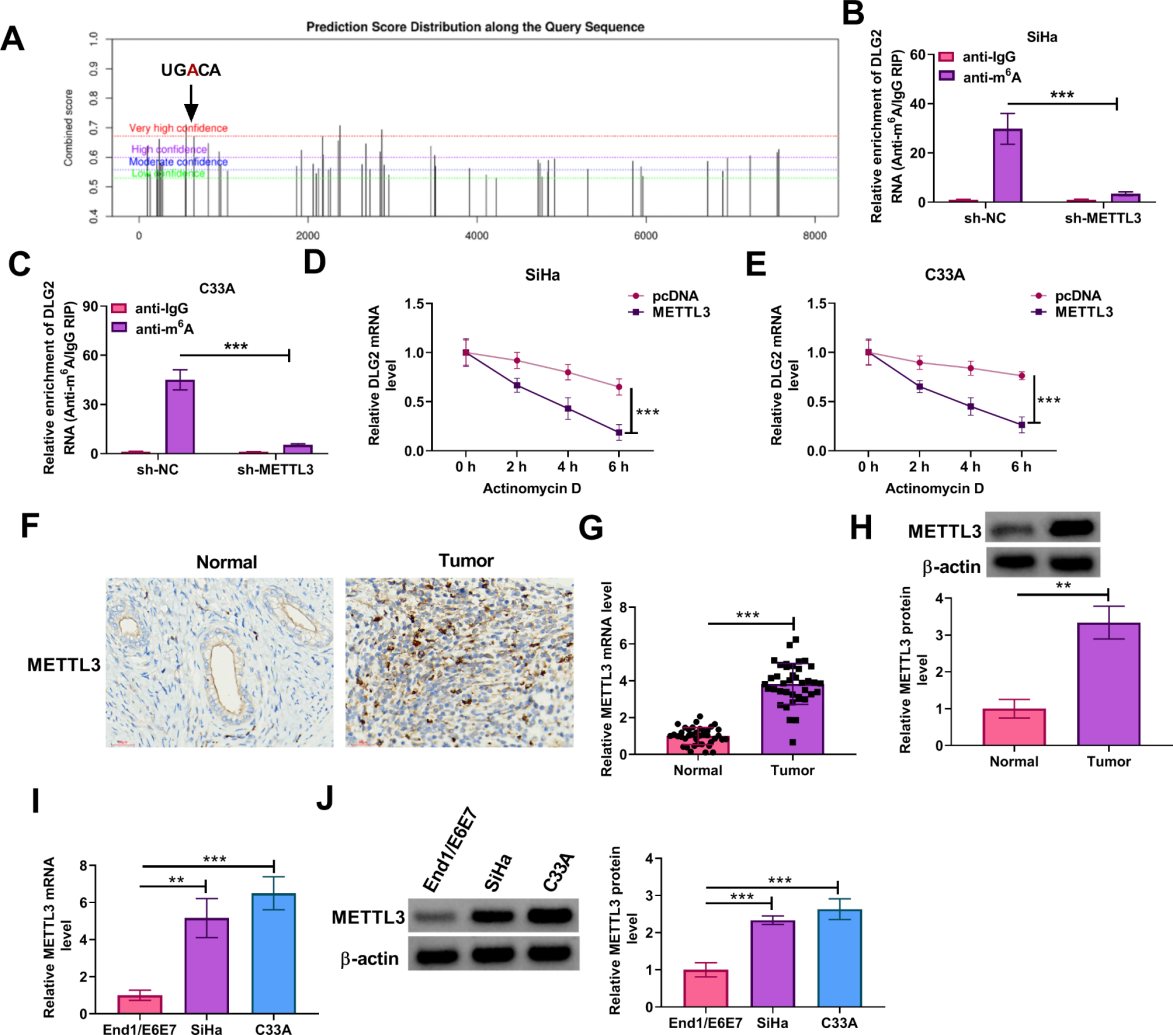
METTL3 destabilized DLG2 mRNA expression in an m6A-dependent manner. (**A**) The RNA-binding proteins site prediction tool SRAMP was used to predict the methylation sites within DLG2. (**B** and **C**) The MeRIP assay was performed to analyze the effect of METTL3 silencing on m6A modification of DLG2 in both SiHa and C33A cells. (**D** and **E**) The effect of METTL3 overexpression on the transcription half-life of DLG2 was analyzed through the Actinomycin D assay. (**F**) The IHC assay was performed to analyze METTL3 protein expression in cervical cancer tissues and normal cervical tissues. (**G**) METTL3 mRNA expression was detected by qRT-PCR in cervical cancer tissues (*N* = 39) and normal cervical tissues (*N* = 39). (**H**) METTL3 protein expression was analyzed by western blotting assay in cervical cancer tissues and normal cervical tissues. (**I**) METTL3 mRNA expression was detected by qRT-PCR in End1/E6E7 cells, SiHa cells, and C33A cells. (**J**) METTL3 protein expression was analyzed by western blotting assay in End1/E6E7 cells, SiHa cells, and C33A cells. **P < 0.01, and ***P < 0.001

The SRAMP, an m6A modification site prediction tool, predicted the presence of methylation sites within DLG2 (Fig. 5A), suggesting that DLG2 may be regulated by its RNA methylation. METTL3 is an m6A “writer” protein that has been reported to promote cervical cancer progression [25]. Herein, our data from the MeRIP assay showed that anti-m6A promoted the m6A abundance of DLG2 mRNA in both SiHa and C33A cells, which was decreased by METTL3 silencing (Fig. 5B and C). Moreover, the transcript half-life of DLG2 was shortened after METTL3 overexpression in both SiHa and C33A cells (Fig. 5D and E). Subsequent data from the IHC assay showed that METTL3 expression was higher in cervical cancer tissues when compared with normal cervical cancer tissues (Fig. 5F). Further, METTL3 expression at mRNA and protein levels was higher in clinical cervical cancer tissues and cells than in normal cervical tissues and cells (Fig. 5G-J). Thus, METTL3 reduced DLG2 expression in an m6A-dependent manner.

**3.6 METTL3 silencing inhibited the malignant progression of cervical cancer cells by regulating DLG2 and the Hippo/YAP signaling**.

**Fig. 6:**
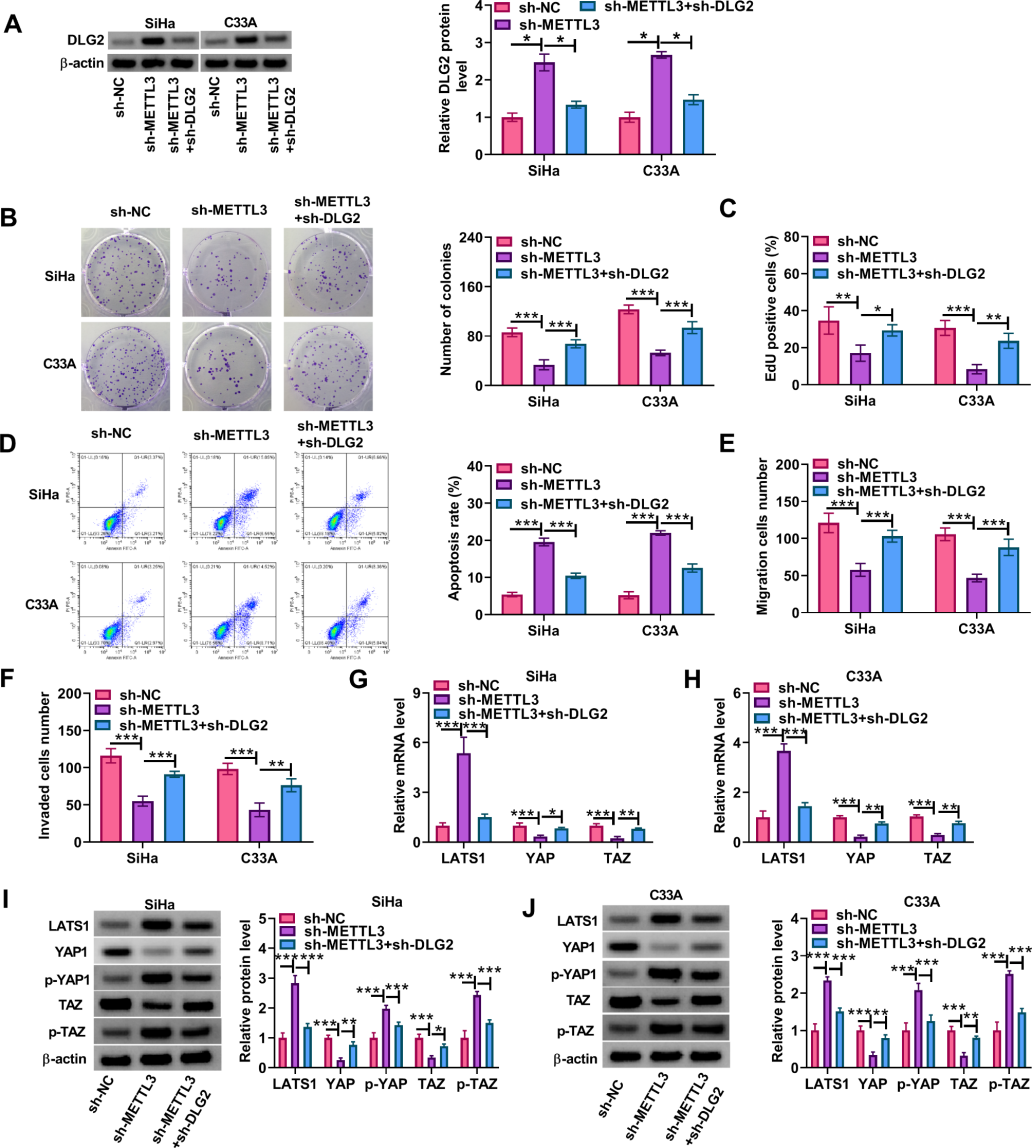
METTL3 silencing inhibited the malignant progression of cervical cancer cells by regulating DLG2 and the Hippo/YAP signaling. Both SiHa and C33A cells were divided into 3 groups, including sh-NC group, sh-METTL3 group, and sh-METTL3 + sh-DLG2 group. (**A**) DLG2 protein expression was detected by western blotting assay. (**B** and **C**) Cell proliferation was analyzed by cell colony formation assay and EdU assay. (**D**) Cell apoptosis was analyzed by flow cytometry. (**E** and **F**) Cell migration and invasion were assessed by transwell assays. (**G** and **H**) The mRNA levels of LATS1, YAP and TAZ were detected by qRT-PCR. (**I** and **J**) The protein levels of LATS1, YAP1, p-YAP, TAZ, and p-TAZ were analyzed by western blotting assays. *P < 0.05, **P < 0.01, and ***P < 0.001

We further analyzed whether DLG2 was involved in the regulation of METTL3 in the malignant phenotypes of cervical cancer cells. To this end, we transfected shRNAs of METTL3 and DLG2 into both SiHa and C33A cells. As shown in Fig. 6A, METTL3 silencing promoted DLG2 protein expression, whereas the effect was relieved after DLG2 knockdown. Subsequently, METTL3 silencing inhibited cell proliferation and induced cell apoptosis, however, these effects were counteracted after DLG2 expression was decreased (Fig. 6B-D). METTL3 knockdown also inhibited cell migration and invasion, whereas the effects were restored after DLG2 depletion (Fig. 6E and F). Comparatively, METTL3-deficient cells showed an increase in LATS1 mRNA expression and a decrease in YAP and TAZ mRNA expression, however, these effects were relieved after DLG2 silencing (Fig. 6G and H). Further, DLG2 depletion restored the METTL3 silencing-induced promoting effects on the protein expression of LATS1, p-YAP and p-TAZ and inhibitory effects on the protein expression of YAP and TAZ (Fig. 6I and J). Thus, METTL3 might promote the malignant progression of cervical cancer cells by decreasing DLG2 expression.

## Discussion

Cervical cancer is a significant global issue that poses a serious threat to female health. The efficacy of treatments such as radiation and chemotherapy is limited and constrained by side effects, thus the overall 5-year survival rate is low. The emergence of targeted and immunotherapy has greatly improved the efficacy of cancer treatment, significantly prolonging the survival of patients [26, 27]. However, issues such as low response rates and primary or secondary drug resistance still need to be addressed urgently. Exploring new treatment targets will help to enhance the effectiveness of cancer therapy and benefit more patients. Thus, an in-depth investigation into the mechanism responsible for cervical cancer progression is of significant importance. We identified a novel mechanism by which METTL3 destabilized DLG2 expression to activate the Hippo/YAP pathway, thus promoting cervical cancer progression.

It has been reported that DLG2 participated in cancer progression. For example, DLG2 inhibited colorectal cancer tumor property through the circ0106714/miR-942-5p/DLG2 pathway [18]. DLG2 knockdown led to forced cell cycle progression and predicted a poor prognosis of neuroblastoma patients [15]. DLG2 inhibited the epithelial-mesenchymal transition of glioma cells by regulating the AKIP1/DLG2 pathway [28]. Another paper revealed that DLG2 could be implicated in the carcinogenesis of cervical cancer caused by HPV [29]. However, there was no data regarding its role in cervical cancer progression. We found a lower DLG2 expression in cervical cancer tissues through the online databases including GEPIA and UALCAN. Moreover, our analysis of clinical cervical cancer samples and normal cervical samples showed the same results. We also detected its lower expression in cervical cancer cells. In addition, DLG2 overexpression inhibited cervical cancer cell proliferation, migration, and invasion and induced cell apoptosis. DLG2 overexpression also delayed tumor formation. The Hippo/YAP pathway can regulate organ size, tissue homeostasis, and tumor progression [30]. This pathway interacted with EGFR signaling and HPV oncoproteins to regulate the malignant development of cervical cancer [31]. Our results showed that DLG2 could inactive the Hippo/YAP pathway. These data demonstrated that DLG2 inactivated the Hippo/YAP pathway to inhibit cervical cancer progression.

Disruption of RNA m6A modification levels is closely associated with tumor progression [32]. METTL3 acts as a methyltransferase and participates in cervical cancer progression. For example, METTL3 enhanced FOXD2-AS1 stability to promote the migration and proliferation of cervical cancer cells [25]. METTL3 maintained the mRNA stability of TXNDC5 to inhibit ER stress, further promoting cervical cancer cell metastasis [33]. Our data showed that METTL3 destabilized DLG2 expression in an m6A-dependent manner. Herein, we found its upregulation in cervical cancer tissues, which was consistent with previous data [34]. Moreover, DLG2 silencing attenuated METTL3 knockdown-induced effects in cervical cancer cells, indicating that the METTL3/DLG2 axis was involved in cervical cancer progression. A previous study revealed that aspartyl-tRNA synthetase 1 antisense 1 (DARS-AS1) recruited METTL3 to mediate its m6A modification in cervical cancer progression [35]. Thus, DARS-AS1 might be required for the modulation of METTL3 in the DLG2/Hippo/YAP signaling in cervical cancer progression.

However, the current study has the following limitations. The analysis is limited by the sample size and representativeness of the cervical cancer tissues and cells used. A larger and more diverse dataset would provide more robust conclusions. Although the inhibition of malignant progression by DLG2 overexpression shows promise, the study’s reliance on in vitro and in vivo models may not fully capture the intricate interactions that occur within the human body. Future research should address this limitation.

Taken together, cervical cancer progression involved a high METTL3 expression, and the elevated METTL3 expression reduced DLG2 release in an m6A-dependent manner to activate the Hippo/YAP pathway, further promoting tumor cell proliferation, migration and invasion and inhibiting cell apoptosis. This study elucidates the specific regulatory role of METTL3 in the progression of cervical cancer, thereby enriching our understanding of the mechanisms underlying the development of this malignancy. In addition, the findings could contribute to the development of new treatment strategies. In particular, the combination of METTL3 inhibitors or DLG2 activators with current cervical cancer therapies has the potential to improve treatment outcomes by leveraging a more comprehensive and targeted approach against the disease. However, clinical trials would be necessary to validate these hypotheses and to determine the safety, efficacy, and optimal use of such combination treatments.

## Electronic supplementary material

Below is the link to the electronic supplementary material.

Supplementary Material 1

## Data Availability

No datasets were generated or analysed during the current study.
