# Supplementary material for: METTL3-dependent DLG2 inhibits the malignant progression of cervical cancer by inactivating the Hippo/YAP signaling
Source: Hereditas. 2025 Jan 25;162:9. doi: 10.1186/s41065-025-00365-z (PMC11762078; doi:10.1186/s41065-025-00365-z)
Supplement: Supplementary file 1 — Supplementary Material 1 [file 41065_2025_365_MOESM1_ESM.pdf]

Fig 1E

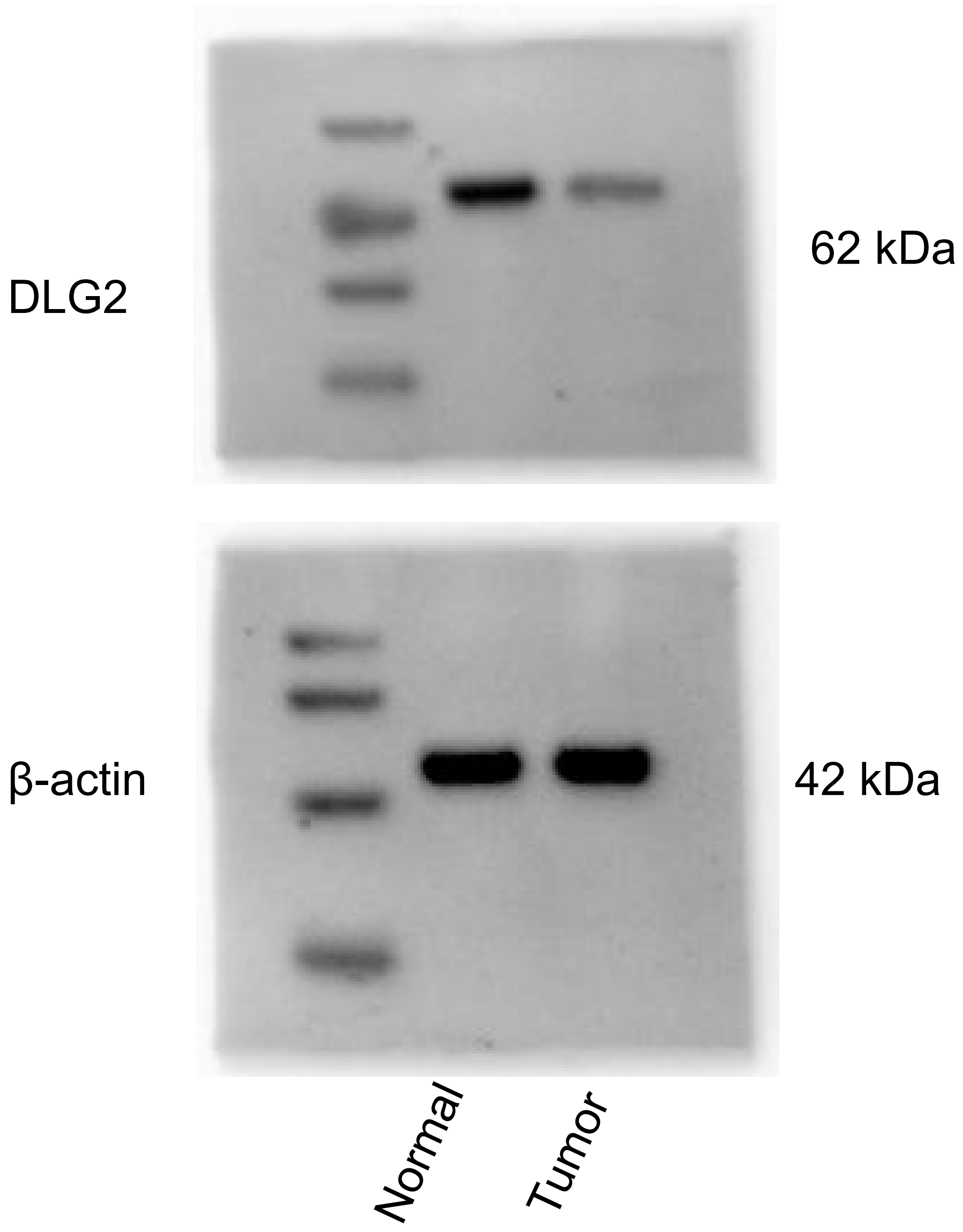
DLG2
62 kDa
β-actin
42 kDa
Normal
Tumor

Fig 1G

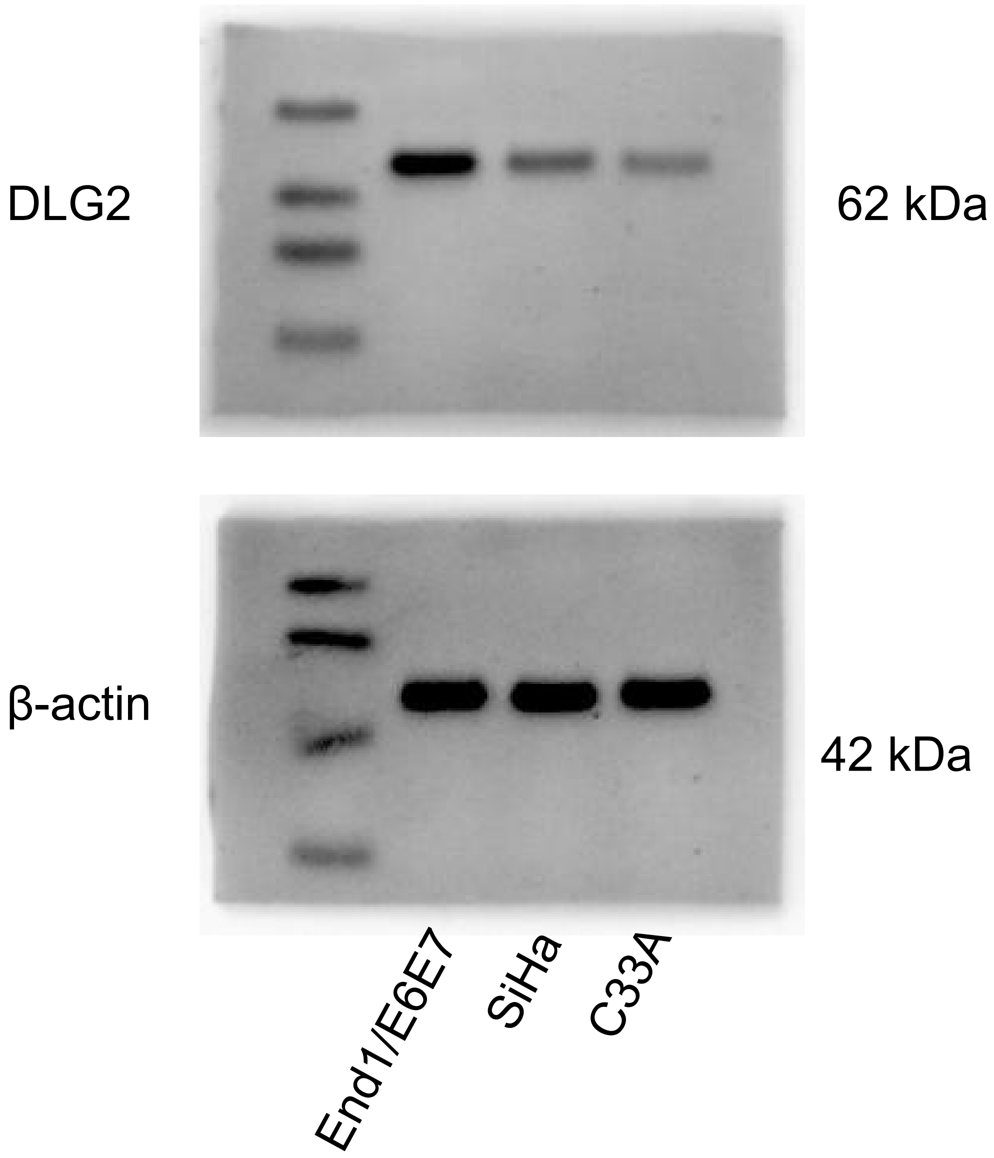
DLG2
62 kDa
β-actin
42 kDa
End1/E6E7
SiHa
C33A

Fig 2A

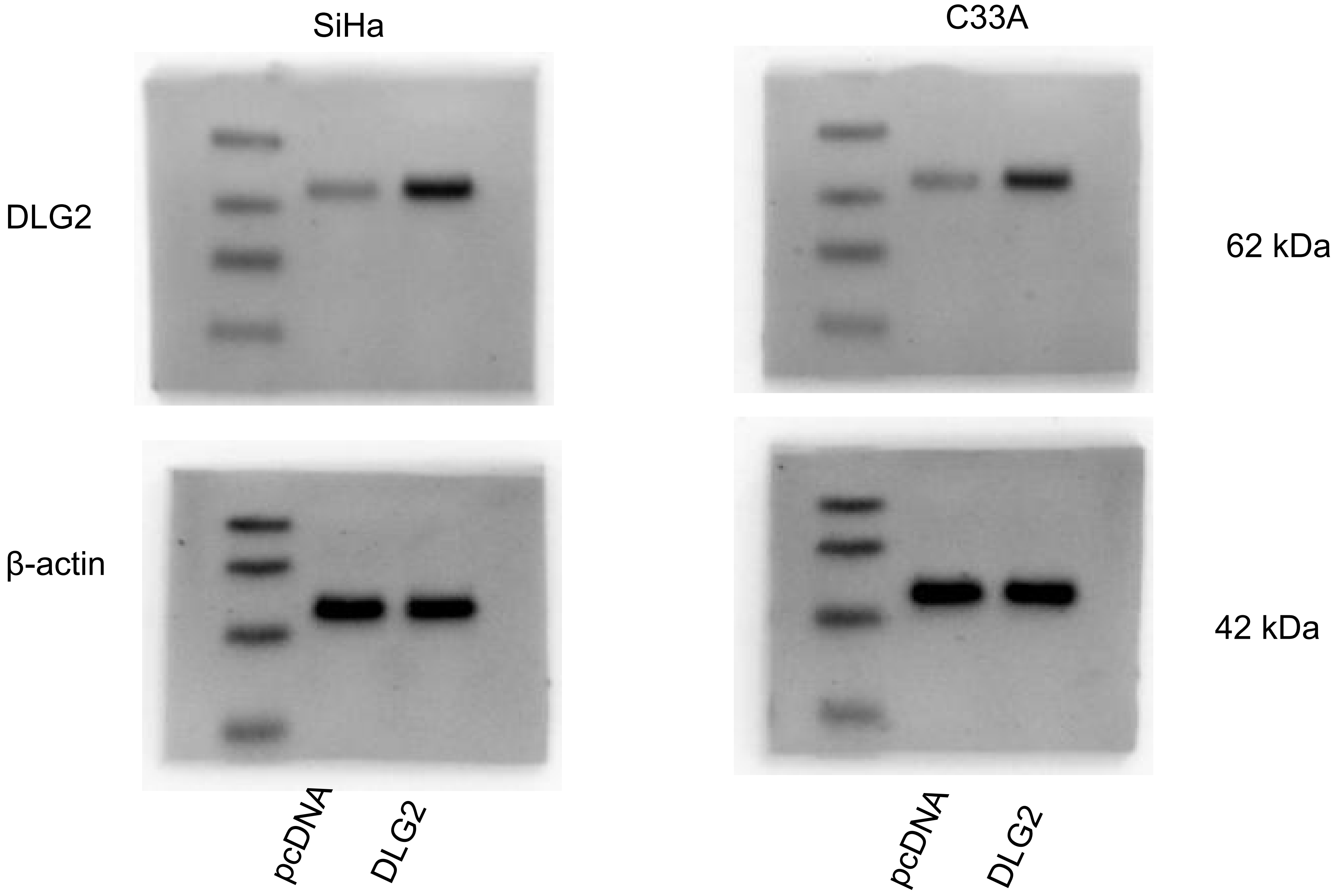

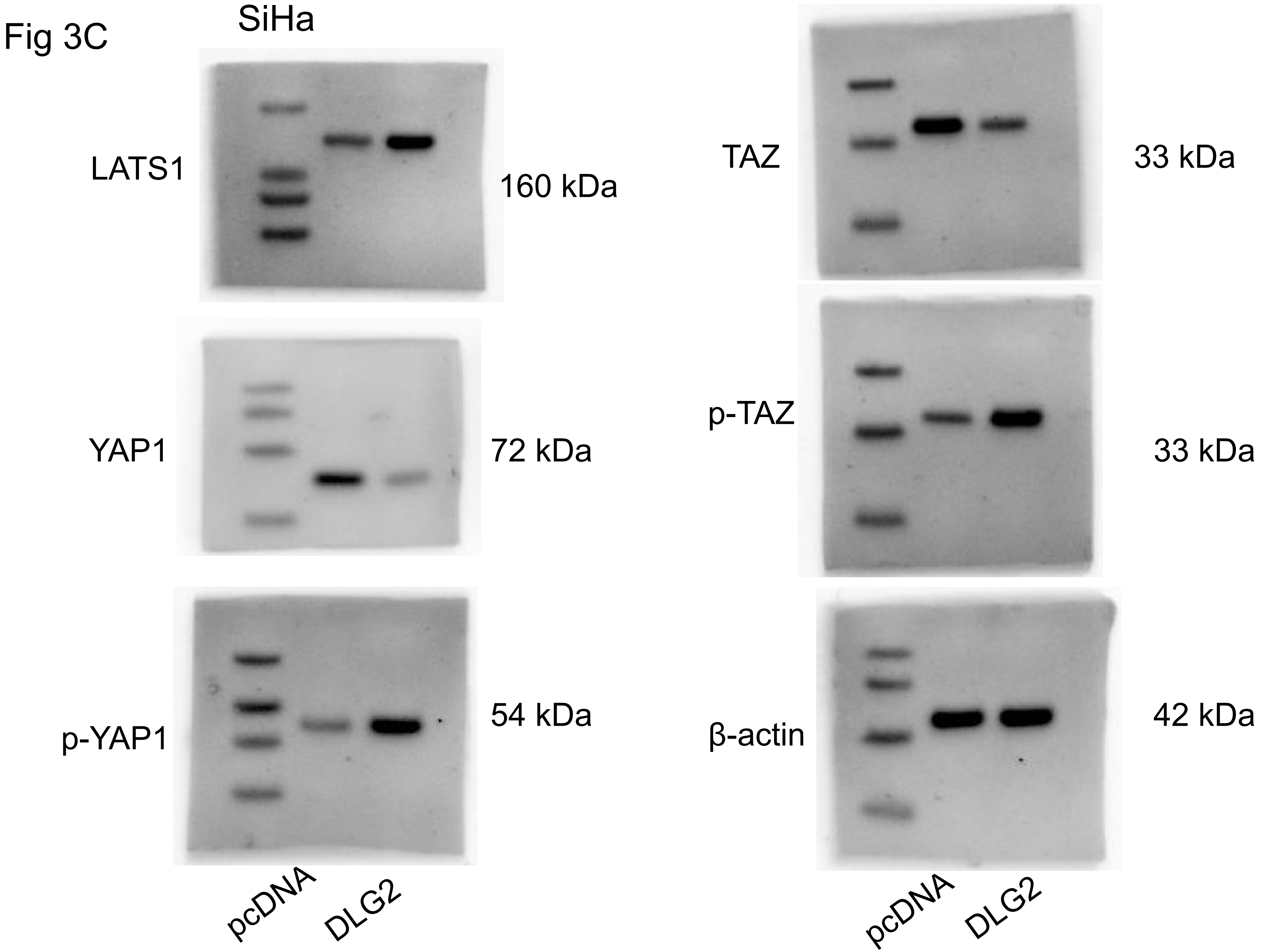
Fig 3C
SiHa
LATS1
160 kDa
YAP1
72 kDa
p-YAP1
54 kDa
TAZ
33 kDa
p-TAZ
33 kDa
β-actin
42 kDa
pcDNA
DLG2
pcDNA
DLG2

Fig 3D

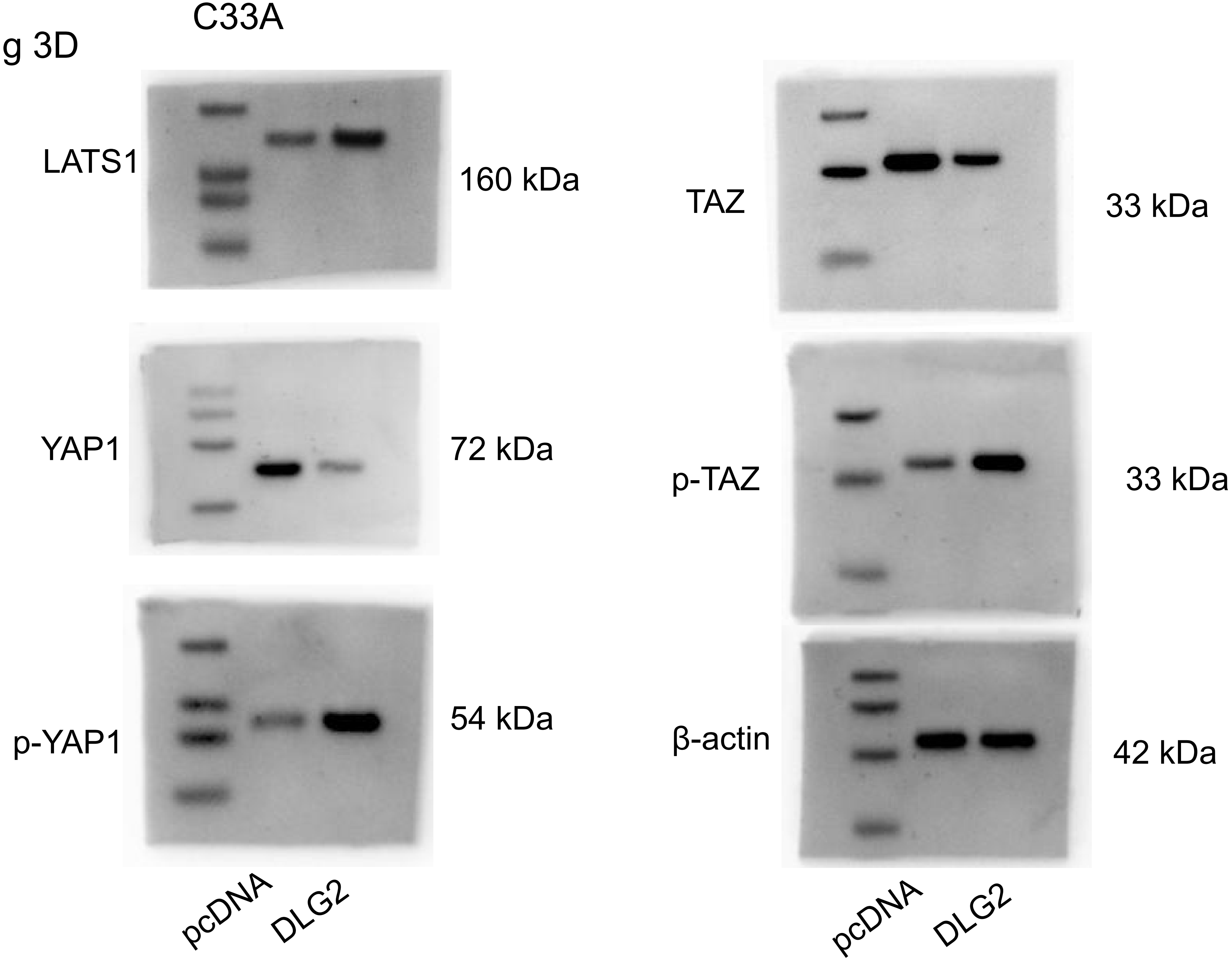
C33A
LATS1
160 kDa
YAP1
72 kDa
p-YAP1
54 kDa
pcDNA
DLG2
TAZ
33 kDa
p-TAZ
33 kDa
β-actin
42 kDa
pcDNA
DLG2

Fig 4C

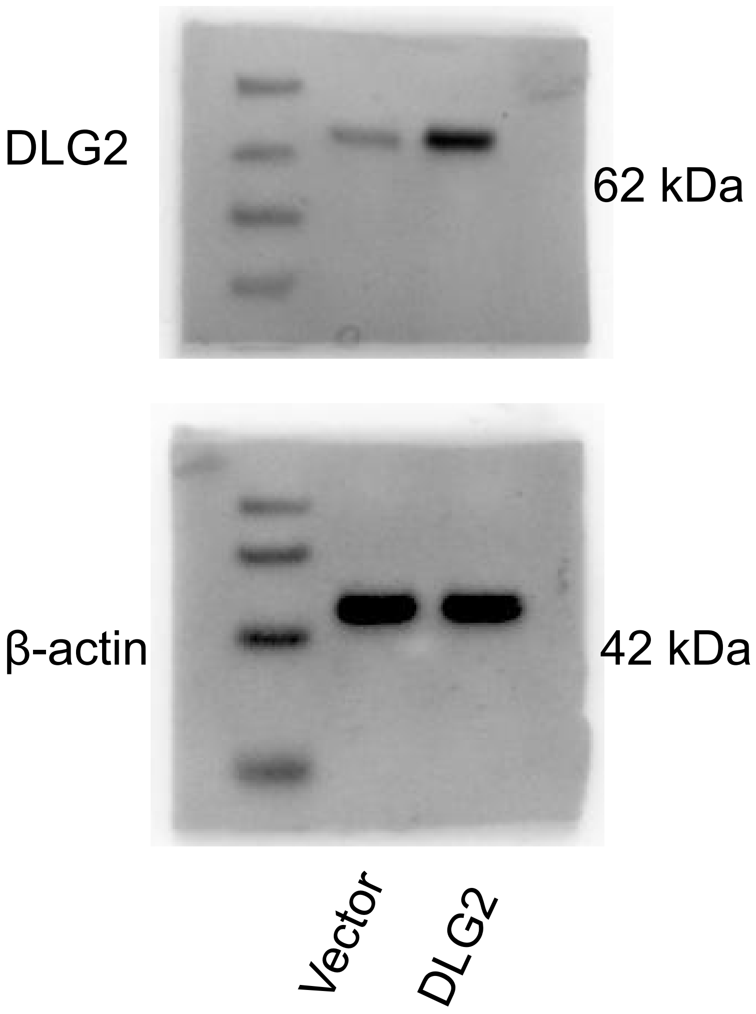

Fig 5H

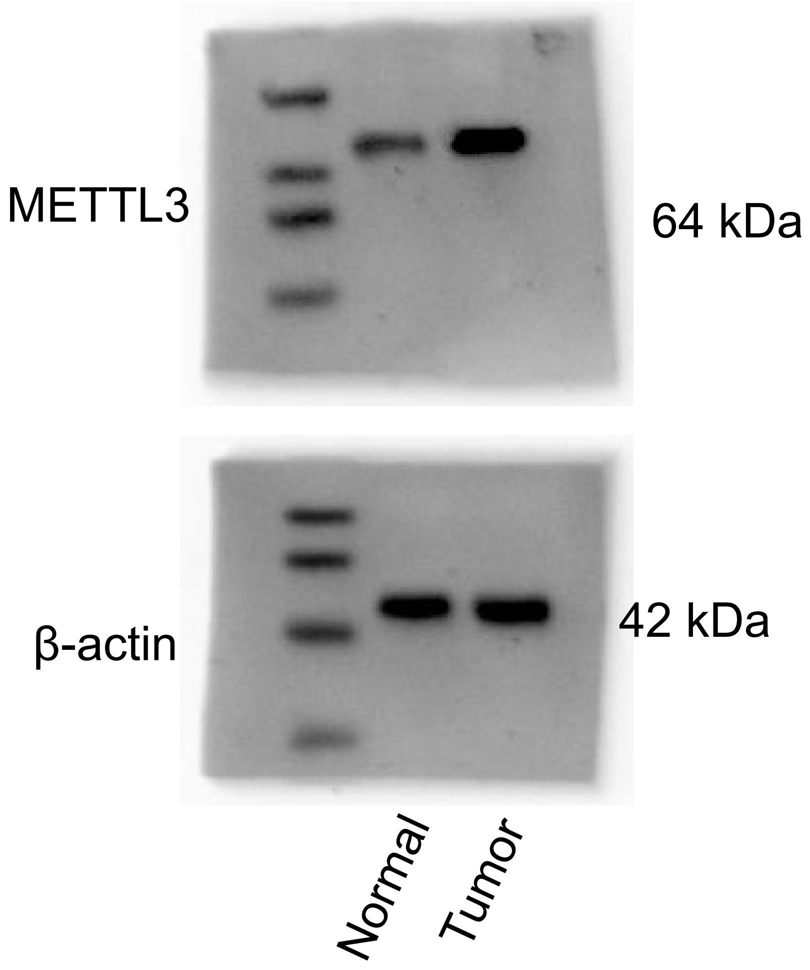

Fig 5J

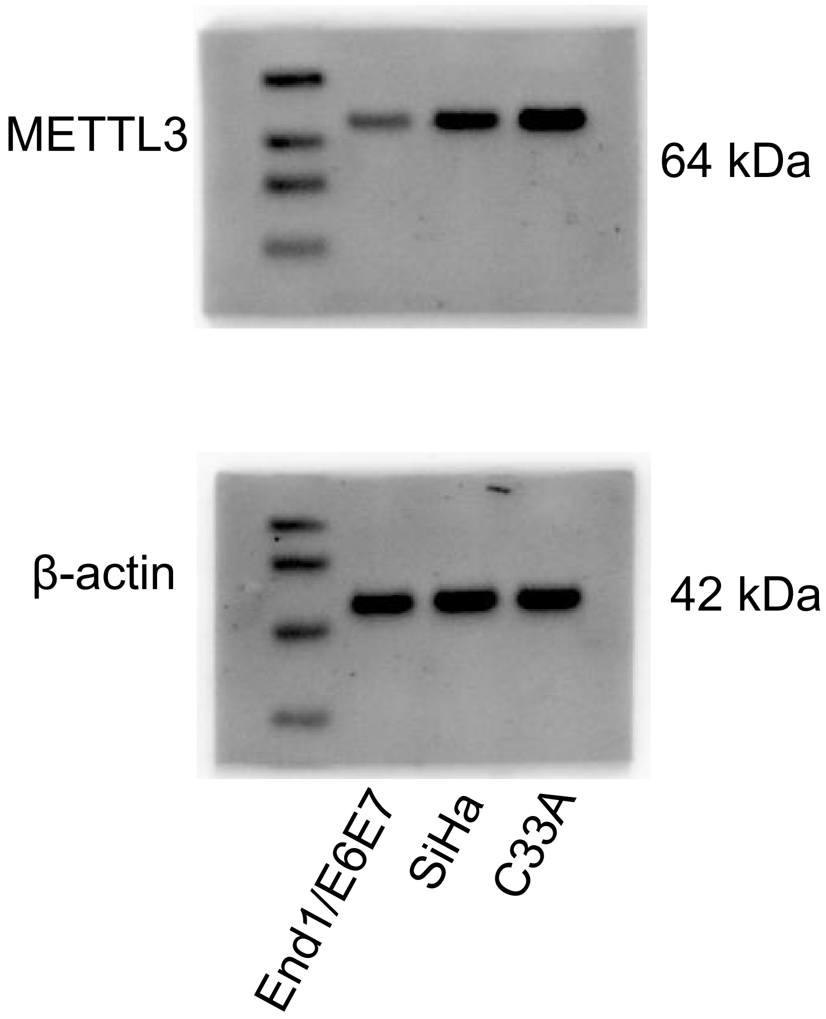

Fig 6A

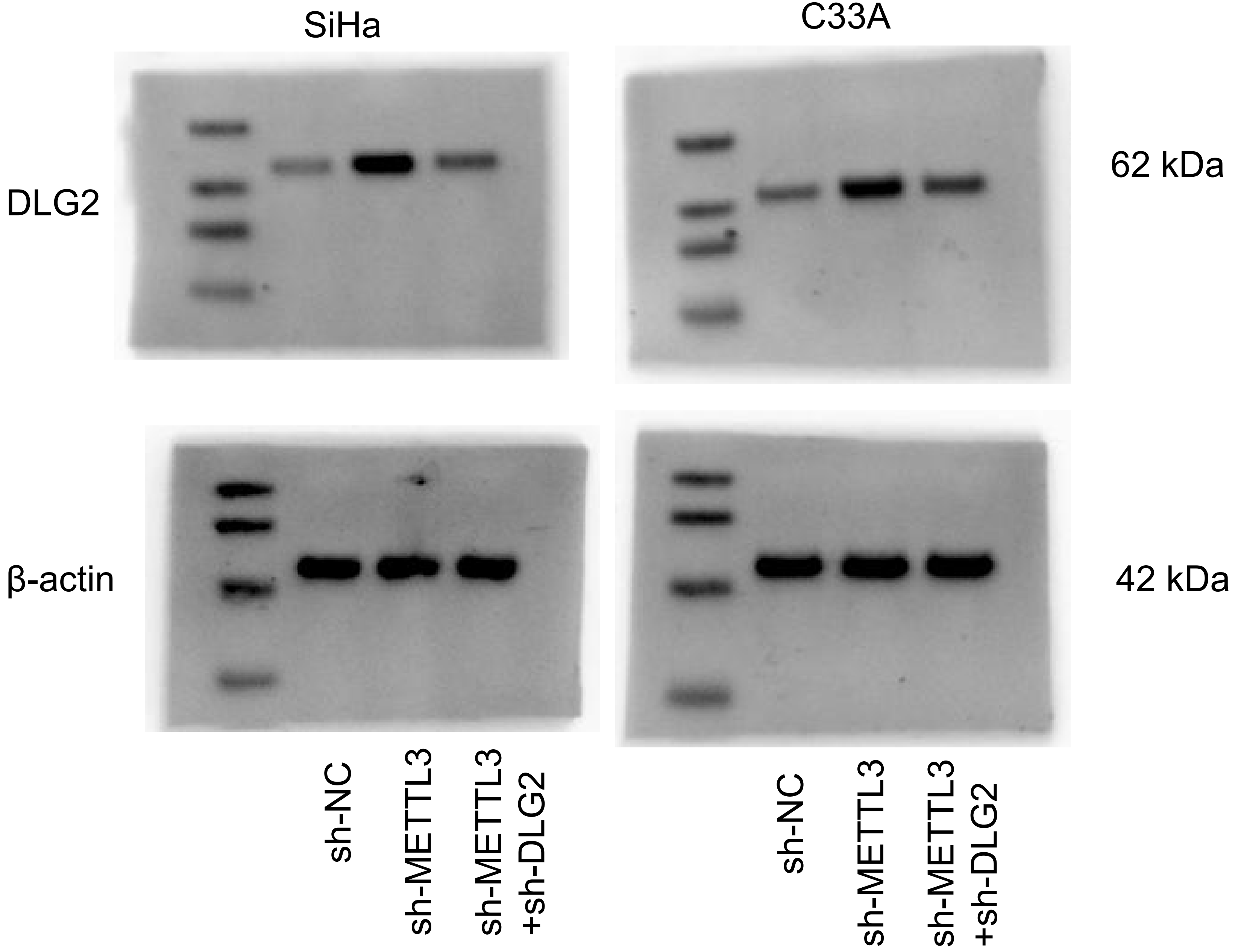
SiHa
C33A
DLG2
β-actin
62 kDa
42 kDa
sh-NC
sh-METTL3
sh-METTL3
+sh-DLG2
sh-NC
sh-METTL3
sh-METTL3
+sh-DLG2

Fig 6I

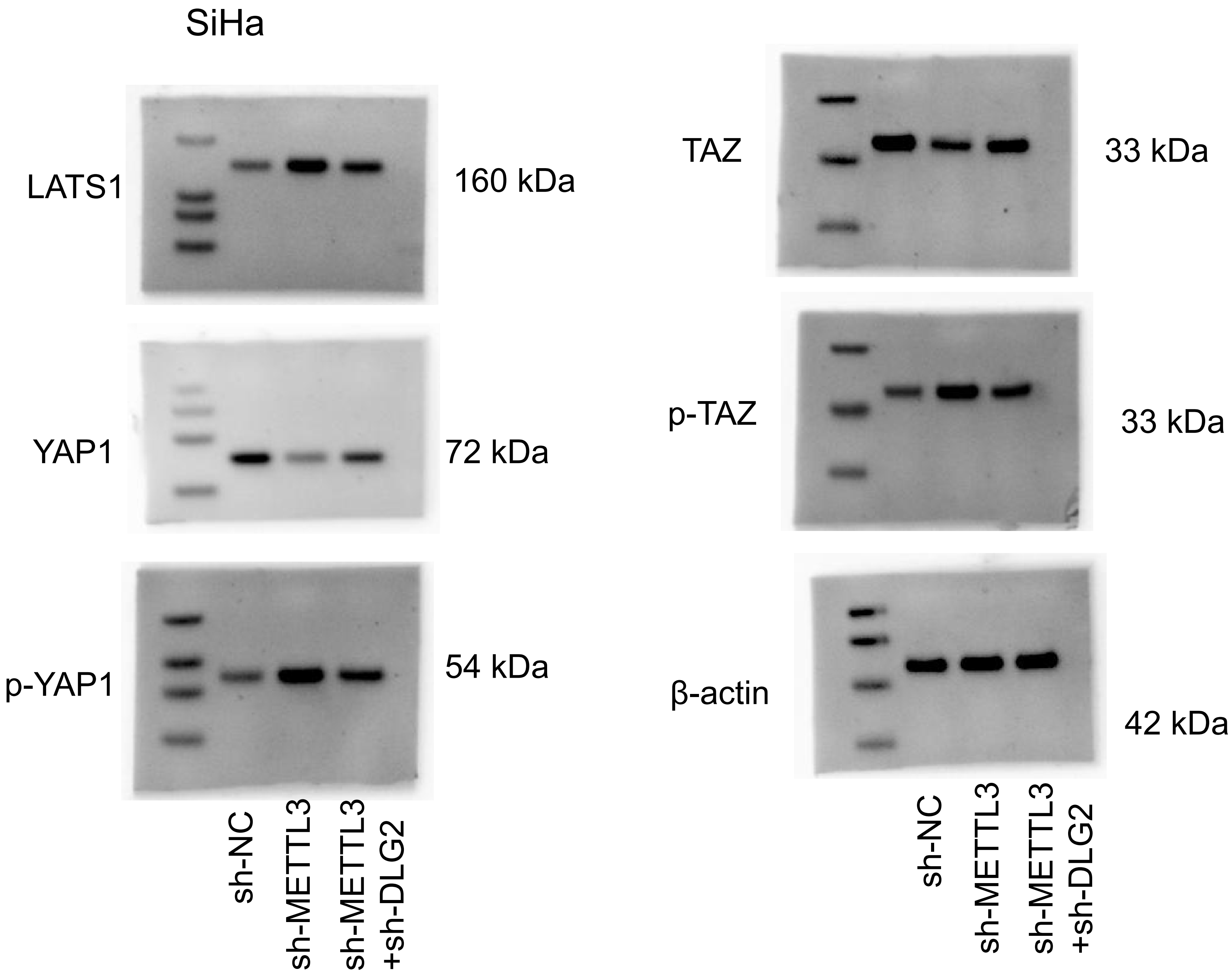
SiHa
LATS1
160 kDa
YAP1
72 kDa
p-YAP1
54 kDa
TAZ
33 kDa
p-TAZ
33 kDa
β-actin
42 kDa
sh-NC
sh-METTL3
sh-METTL3
+sh-DLG2
sh-NC
sh-METTL3
sh-METTL3
+sh-DLG2

Fig 6J

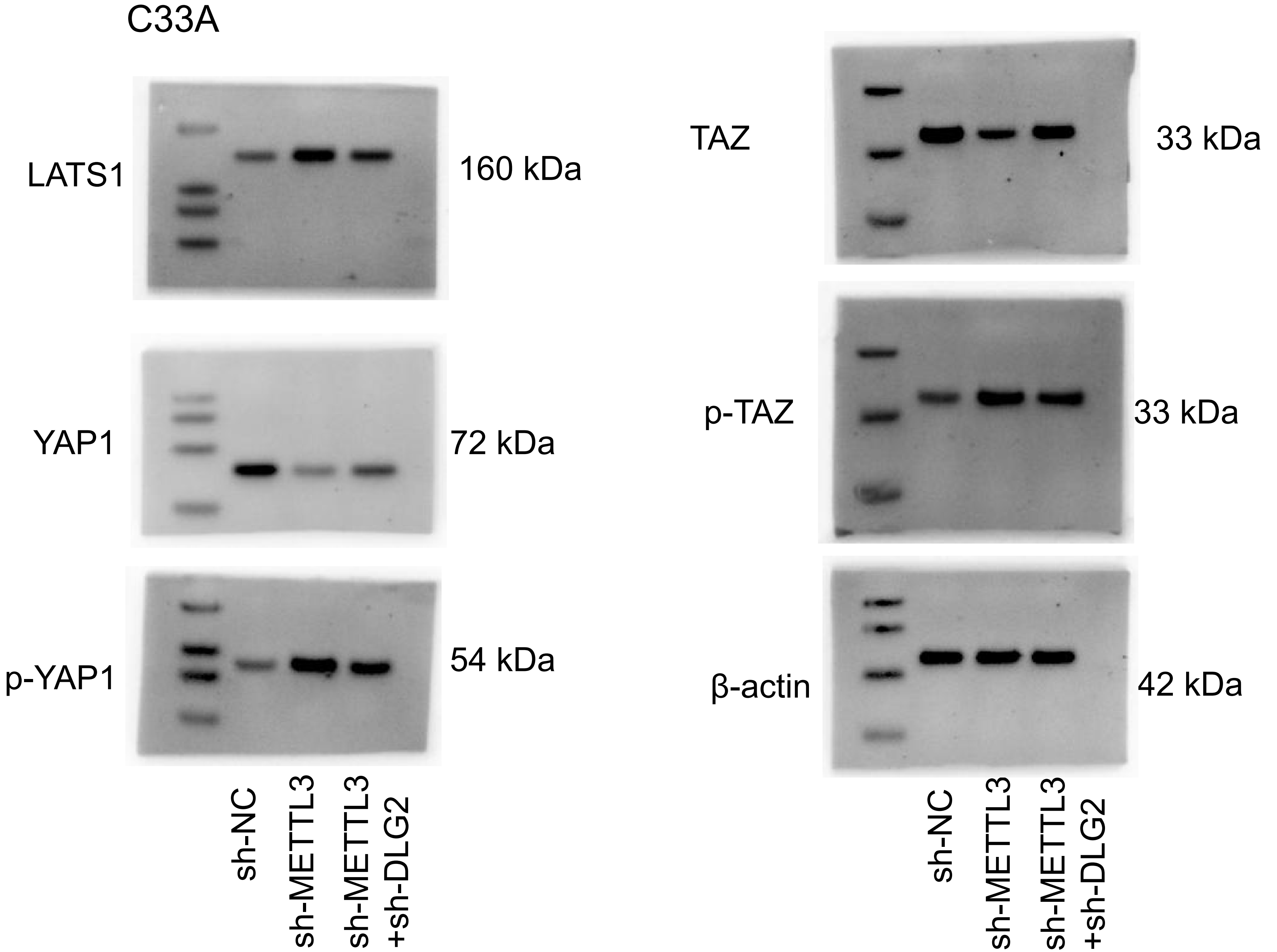

C33A
LATS1
160 kDa
YAP1
72 kDa
p-YAP1
54 kDa
TAZ
33 kDa
p-TAZ
33 kDa
β-actin
42 kDa
sh-NC
sh-METTL3
sh-METTL3 +sh-DLG2
sh-NC
sh-METTL3
sh-METTL3 +sh-DLG2
